# Ethnicity-specific distribution of TRPM8 gene variants in Eurasian populations: signs of selection

**Published:** 2020-05

**Authors:** T.A. Potapova, A.G. Romashchenko, N.S. Yudin, M.I. Voevoda

**Affiliations:** Institute of Cytology and Genetics of Siberian Branch of the Russian Academy of Sciences, Novosibirsk, Russia; Institute of Cytology and Genetics of Siberian Branch of the Russian Academy of Sciences, Novosibirsk, Russia; Institute of Cytology and Genetics of Siberian Branch of the Russian Academy of Sciences, Novosibirsk, Russia Novosibirsk State University, Novosibirsk, Russia; Institute of Cytology and Genetics of Siberian Branch of the Russian Academy of Sciences, Novosibirsk, Russia Federal Research Center of Fundamental and Translational Medicine, Novosibirsk, Russia Novosibirsk State University, Novosibirsk, Russia

**Keywords:** TRPM8 gene, haplotypes, Eurasian human populations, alternative start codons

## Abstract

The TRPM8 gene encodes the ion channel, which is a cold receptor in afferent neurons of the mammalian somatosensory system. We studied the frequency of haplotype distribution from six SNPs in the TRPM8 gene in Eurasian human populations, including Russians, Kazakhs and Chukchi. Four of the six SNPs are located in exon 7 (rs13004520, rs28901637, rs11562975, rs17868387), rs7593557 is in exon 11. These exons encode parts of the N-terminus, which is necessary for channel functioning in the plasma membrane of neurons. The rs11563071 is in exon 23 encoding part of the C-terminus. The primary difference in population distribution of haplotypes determines the SNP from exon 11 which leads to Ser419Asn substitution in protein. The most pronounced differences in the patterns of diversity and frequencies of haplotypes were observed between Chukchi and Russians. The frequency of major H1 haplotype encompassing the 419Ser gene variant differs in examined populations; 0.738 (Russians), 0.507 (Kazakhs) and 0.337 (Chukchi), p < 0.001. The TRPM8 gene variants encoding 419Asn and carrying the minor alleles of rs28901637 (P249P) and rs11562975 (L250L) in exon 7 are characteristic of Asian populations. The frequency of all 419Asn variants in Chukchi is comparable to that in Africans, however, the minor allele frequencies of rs28901637, rs11562975 in Africans is low. Apparently in the process of human colonization of Eurasia, minor alleles of these SNPs diverged depending on rs7593557 structure in exon 11. We analyzed sequences of five TRPM8 mRNA isoforms extracted by researchers from different tissues. Sequence analysis demonstrates that they are transcribed from major H1 variant of the TRPM8 gene but contain different translation start codons, which are generated by alternative splicing from pro-mRNA.

## Abstract (ru)

Ген TRPM8 кодирует ионный канал, который является холодовым рецептором в афферентных нейронах соматосенсорной системы млекопитающих. Мы изучили распределение частот гаплотипов из шести ОНП гена TRPM8 в евразийских популяциях человека, включая русских, казахов и чукчей. Четыре из шести ОНП расположены в экзоне 7 (rs13004520, rs28901637, rs11562975, rs17868387), ОНП rs7593557 находится в экзоне 11. Эти экзоны кодируют фрагменты N-терминального домена, необходимого для функционирования канала в плазматической мембране афферентных нейронов. ОНП rs11563071 расположен в экзоне 23, кодирующем фрагмент С-терминального домена канала. Основное различие в популяционном распределении гаплотипов определяет ОНП из экзона 11, обусловливающий Ser419Asn замещение в белке. Контрастные различия в многообразии и частотах гаплотипов наблюдали между популяциями чукчей и русских. Частоты основного гаплотипа Н1, относящегося к 419Ser вариантам гена TRPM8, существенно различались в изученных популяциях: 0.738 у русских, 0.507 у казахов, 0.337 у чукчей (p < 0.001). Для азиатских популяций характерны варианты гена TRPM8, кодирующие 419Asn и содержащие минорные аллели ОНП rs28901637 (P249P) и rs11562975 (L250L) в экзоне 7. Суммарная частота таких гаплотипов у русских составляет 0.032, по сравнению с 0.142 у казахов и 0.358 у чукчей (p < 10–3 для обоих сравнений). Частота всех 419Asn вариантов у чукчей сопоставима с таковой африканцев, однако частота минорных аллелей rs28901637 и rs11562975 у африканцев низкая. По-видимому, в процессе колонизации человеком Евразии минорные ал лели этих ОНП дивергировали в зависимости от структуры rs7593557 в экзоне 11. Нами проанализированы последовательности пяти изоформ мРНК гена TRPM8, выделенных исследователями из разных тканей. Пока- зано, что они транскрибированы c основного варианта H1 гена TRPM8, но содержат различные старт-кодоны трансляции, генерированные альтернативным сплайсингом про-мРНК.

## Introduction

The gene TRPM8 encodes a subunit of Ca^2+^-permeable nonselective cation channel, belonging to the TRPM (transient receptor potential melastatin) subfamily of TRP domaincontaining proteins (Tsavaler et al., 2001). TRP channels are formed by oligomerization of subunits sharing common structural features, including six putative transmembrane segments (S1–S6), a pore loop linking segments S5 and S6, and cytoplasmic N- and C-terminal (Ramsey et al., 2006). The majority of TRP proteins carry a conserved TRP box (‘VWKFQR’ in TRPM channels) in the C-terminal domain, adjacent to the S6 segment. Many of these proteins, including TRPM8, are involved in Ca^2+^ homeostasis in response to extracellular and intracellular physical and chemical factors. TRPM8 expression has been observed in somatic afferent neurons, myocytes, epithelial cells of the lung, bronchi, prostate, bladder and others (Sabnis et al., 2008; Babes et al., 2011). The modulation of TRPM8 protein activity is coupled with basic biochemical and physiological processes related to thermal sensitivity, proliferation, and apoptosis (Zhang, Barritt, 2006; Yee, 2015).

Three types of TRPM8 polypeptides potentially able to form Ca^2+^ channels by tetramerization have been described. The full-length variant of 1104 amino acids (aa) identified in sensory neurons contain the long N-terminal sequence (693 aa), six transmembrane segments and C-terminal domain with TRP box (Latorre et al., 2011). This channel is able to respond to changes in ambient temperature (threshold of ~22–34 °C). Another TRPM8 isoform of 1054 aa, with a rearranged N-terminal domain, was identified in prostate tumor cells (Lis et al., 2005). In addition, a truncated variant (304 aa), lacking the entire N-terminal sequence and the first two transmembrane segments (S1 and S2), but retaining parts of the voltage-dependent sensory module (S3 and S4), the pore-forming components (S5 and S6 and the loop between them), and the C-terminal domain, was identified in human epithelial cells of the bronchi and lung (Sabnis et al., 2008). Full-length TRPM8 is located to the plasma membrane, while the truncated variant is located in the endoplasmic reticulum membrane and is associated with the release of Ca^2+^ ions from intracellular stores (Bidaux et al., 2007).

Studies clarifying the contribution of the N-terminal domain to the TRPM8 channel function have shown that the first 40 amino acids of full-length TRPM8 modulate sensitivity of the protein to cold and menthol, and the region between residues 40 and 60 is involved in trafficking of the protein to the plasma membrane (Phelps, Gaudet, 2007; Bidaux et al., 2012; Pertusa et al., 2014). It is also an essential element in ensuring the proper folding and assembly of TRPM8.

In this study, we evaluated the distribution of the alleles at five SNPs located in TRPM8 gene exons 7 and 11 in geographically dispersed Eurasian human populations in order to clear up the potential importance of the N-domain parts encoded by these modifiable exons. The composition and localization of the SNPs assayed in the TRPM8 gene are unique. Four of six SNPs are densely clustered in exon 7; three in successive codons (encoding P249P, L250L, and Y251C), and the fourth in the codon but one upstream (encoding R247T). The SNP (S419N) in exon 11 and two SNPs (R247T and Y251C) from exon 7 can potentially influence on function of the TRPM8 channel by altering the protein structure and accessibility of these sites to post-translational modification. The two SNPs (P249P and L250L) in exon 7 and the SNP (encoding V1058V in the C-terminal domain) in exon 23 cannot directly influence on protein structure. However, recurrent emergence of the minor alleles of these SNPs in different TRPM8 gene variants suggests that they may have influence on gene expression regulation.

## Materials and methods

Human populations that have resided in geographically dispersed territories in Eurasia were examined, including Russians (N = 170, Novosibirsk), Kazakhs (N = 119, Kosh-Agach district, the Altai Republic) and Tundra Chukchi (N = 80, Kanchalan settlement, Chukotka Autonomous district). Ethnicity of individuals was determined by special questioning with elucidation of a nationality of the ancestors (at least in three generations). Blood samples were collected from unrelated representatives of the ethnic group. All subjects gave their informed consent for participation in the experiment. The work was approved by the Bioethical Committee of the Institute of Cytology and Genetics of Siberian Branch of the Russian Academy of Sciences.

The general characteristics of the assayed SNPs are listed in Table 1.

**Table 1.**
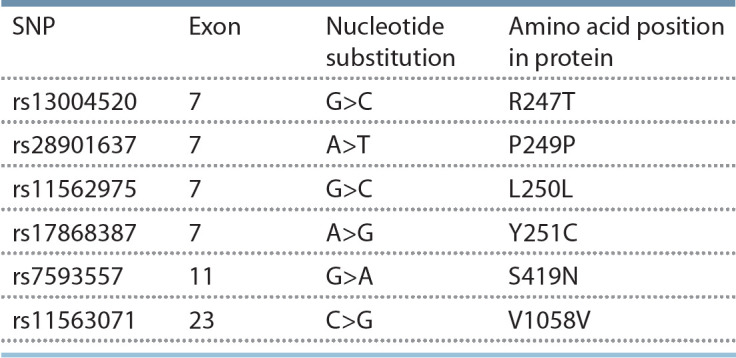
TRPM8 single nucleotide polymorphisms included in this study Note. Amino acid positions are given to the full-length protein from neurons.

The information about variants and reference sequences (NM_024080.4 and BC143819.1) were extracted from dbSNP (https://www.ncbi.nlm.nih.gov/snp/) and GenBank (https://www.ncbi.nih.gov/genbank/).

Genomic DNA from blood samples was isolated by phenolchloroform extraction. DNA was genotyped by polymerase chain reaction. The details of the assay (primers and PCR parameters) developed for genotyping were described previously (Potapova et al., 2014). Primers for rs17868387 fragment (200 nucleotides) was following: 5ʹ-tgtgtggttgttgttcaggagat- 3ʹ (A allele), 5ʹ-tgtgtggttgttgttcaggagac-3ʹ (G allele), 5ʹ-aaagctg ggaggaggaataatgt-3ʹ (total). The annealing temperature of the primers was 60 °C.

Haplotype variants, coefficients of linkage disequilibrium (Dʹ) between the alleles of SNPs, fixation index Fst were determined using Arlequin ver. 3.5.2. The F_st_ was calculated on the basis of haplotype distributions. The level of significance ( p_χ2_) of the differences between haplotype groups was assessed using SPSS 11.5 software.

## Results

The distribution of haplotypes at six SNPs within the TRPM8 gene in individuals from Russian (European), Kazakh (Central Asians) and Chukchi (Eastern Asians) populations, who have been resident in different climatic and geographical zones of Eurasia, was examined in this study. Twenty two haplotypes with frequencies exceeding 0.001 at least in one of the three populations were identified (Table 2).

**Table 2.**
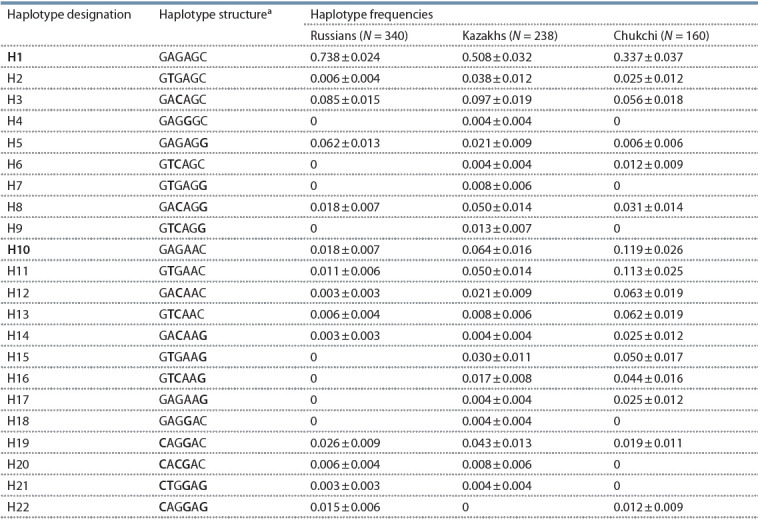
TRPM8 haplotypes in Russian, Kazakh, and Chukchi populations Notes. a The SNP numeration in haplotypes are as follows: 1 – rs13004520: G>C, (R247T); 2 – rs28901637: A>T, (P249P); 3 – rs11562975: G>C, (L250L); 4 – rs17868387: A>G, (Y251C); 5 – rs7593557: G>A, (S419N); 6 – rs11563071: C>G, (V1058V). N – number of chromosomes.

The haplotypes can be categorized into two main groups, according to nucleotide substitutions of the rs7593557 SNP (exon 11) at position 5 in each of population samples. The G allele of this SNP leads to formation of the 419Ser codon in the mRNA. The 419Ser group includes the major H1 haplotype and H2–H5 with singleton substitutions at one of the five positions relative to the H1. The H6–H9 haplotypes differ by the allele combinations of the SNPs (P249P, L250L, V1058V) at positions 2, 3 and 6.

The founder haplotype H10 and its derivatives (H11–H22) carry the A allele (rs7593557, exon 11) at position 5 forming the 419Asn codon. The haplotypes constituting this group have additional substitutions at one or several positions. The H11–H17 subgroup includes haplotypes with minor allele combinations of rs28901637, rs11562975 and rs11563071. The H18–H22 haplotypes contain a substitution at position 4 (rs17868387, exon 7), which leads to a Tyr→Cys replacement. Presumably, this is a recurrent mutation, unrelated to haplotype H4, and variant H18 is the founder haplotype for H19–H22 subgroup. In additional, this haplotypes contain a substitution at position 1 (rs13004520, exon 7), leading to an Arg→Thr replacement. Thus, 419Asn H19-H22 haplotypes contain three substitutions that can affect the TRPM8 protein structure. The linkage disequilibrium (LD) between minor alleles of rs13004520 and rs17868387 SNPs was observed in all three populations (Dʹ = 1). The minor alleles of this SNPs was also linked to rs7593557 A allele from exon 11 (Dʹ = 1). In Kazakhs the rs17868387 G allele was observed in combination with both rs7593557 alleles (Dʹ = 0.9104). The presence of the nucleotide substitutions at positions 2, 3, and 6 (rs28901637, rs11562975, rs11563071, respectively) in different haplotype subgroups (H6–H9, H10–H17 and H19–H22) suggests that their appearance could be repeatedly.

Comparison among the populations revealed considerable differences in the frequencies of the H1 haplotype, with 0.738 in Russians versus 0.508 and 0.337 in Kazakhs and Chukchi, respectively (Table 3).

**Table 3.**
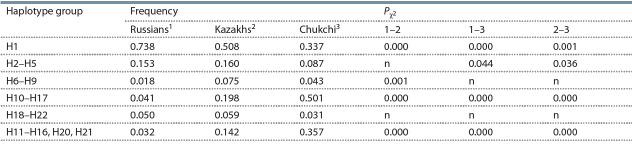
Frequencies of TRPM8 haplotype groups in Russian, Kazakh and Chukchi populations and significance of the inter-population differences Note: n – differences are unreliable.

The 419Ser H2–H5 singleton variants, except H3, have its own specific distribution in the examined populations. The H2 haplotype is present at very low frequency in the Russian population. The frequency of the haplotype H5 is higher in Russians in comparison with Asian populations, while haplotype H4 is absent in the Russian and Chukchi populations and present at a very low frequency in Kazakhs (0.004). The total frequency of the H2–H5 singleton variants is lower in Chukchi in comparison with Russian and Kazakh populations. The 419Ser H6-H9 subgroup demonstrates the more haplotype variety and total haplotype frequency (0.075 %) in Kazakhs compared with Russians (0.018).

It is evident that Russians differ drastically from Asian populations in relation to Asn TRPM8 gene variants, with the exception of H19–H22, in which is observed similar frequencies in both European (0.050) and Asian (0.059 and 0.031) populations. Subgroup H10–H17 is the more heterogeneous (see Table 2) and demonstrates the most pronounced differences between populations in total frequency values: 0.041 in Russians versus 0.198 in Kazakhs, and 0.501 in Chukchi (see Table 3). In the Russian sample some variants in this subgroup are either absent or present with considerably lower frequencies compared to the Chukchi and Kazakh populations. The pattern of haplotype diversity observed in Chukchi is opposite to that in the Russian population; with relatively high frequencies of haplotypes H10 and H11 (0.119 and 0.113 respectively) and similar frequencies among the remaining haplotypes (H12–H17) in the range 0.025–0.063. The presence of minor alleles at positions 2 and 3 in the majority of haplotypes in this subgroup correlates with the relatively higher frequency of these variants in the Asian populations.

The total frequencies of haplotypes containing the minor alleles of the rs28901637 and rs11562975 SNPs (7 exon), which have no direct functional effect on the protein, show characteristic differences among the Russian and Asian populations. The total frequency of haplotypes containing the minor T allele at position 2 (rs28901637) in Russians is very low (0.026), relative to 0.306 and 0.172 in the Chukchi and Kazakh populations, respectively (see Table 2). The Russian population also has a very low frequency of variants containing a substitution at position 3 (rs11562975), except haplotype H3, with a total frequency (without H3) of 0.036, versus 0.125 in Kazakhs and 0.238 in Chukchi ( p < 10^–3^ for both comparisons). The total frequencies of 419Asn variants with minor rs28901637 and rs11562975 alleles in Russians was 0.032, compared with 0.142 in Kazakhs and 0.357 in Chukchi (see Table 3).

In addition Kazakh population shows a clear tendency to increase the frequency of H1 haplotype similar to the Russian population, and a decrease in the total frequency of haplotypes belonging to 419Asn subgroup H10–H17 in comparision with Chukchi. Possible, it is a consequence of their common evolutionary history in the territory of Eurasia. The highest inter-population difference observed between Russian and Chukchi populations (Fst = 0.1442, p < 10^-5^). The estimated F_st_ values generated by comparisons of Russians versus Kazakhs and Kazakhs versus Chukchi were 0.0498 and 0.0242, respectively ( p < 10^–5^ for both comparisons).

## Discussion

According to the current paradigm, anatomically modern humans have spread out of Africa over 60,000–80,000 years ago, following different routes to colonize Europe and Asia (Stoneking, Delfin, 2010; Stewart, Stringer, 2012). Respectively, to assist in interpretation of our results, we considered additional data describing the MAFs distribution of the six SNPs within the TRPM8 gene included in this study in African, European, Chinese, and Japanese populations, extracted from the dbSNP (Table 4).

**Table 4.**
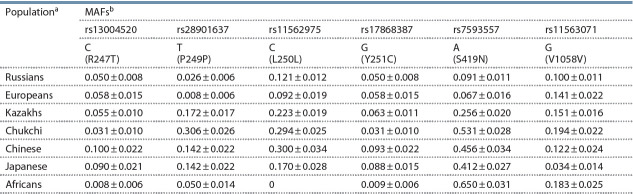
Minor allele frequencies of TRPM8 gene SNPs in African and Eurasian populations

Notes. ^a^ The MAFs in Europeans (CEU), Chinese (CHB), Japanese (YPT), and Africans (YRI) are extracted from dbSNP; ^b^ MAF – minor allele frequency.

This analysis produced the following unambiguous inferences: (1) haplotypes comprising the rs7593557 A allele (exon 11) are widely distributed among Africans and Eastern Asians (Chinese, Japanese, and Chukchi), in comparison with Russians and Europeans. (2) There is absent the rs11562975 minor C allele (exon 7) in the African population and it’s present at frequencies of 0.170–0.300 in Asian haplotypes. A similar situation is observed for the rs28901637 T allele (exon 7), which occurs at a relatively low frequency in African and European populations and at higher frequencies (0.142–0.306) in Asians. (3) No pronounced continental or ethnic specificity in the distribution of the haplotypes containing the rs11563071 minor G allele (exon 23) in the examined populations was detected. The exception is the Japanese population, in which the MAF at this SNP comprises 0.034, compared with 0.183–0.194 in Africans and Chukchi, and 0.100–0.141 in Russians and others Europeans. Consequently, it may be supposed that both of the Ser and Asn TRPM8 gene variants were present in the ancestral population of anatomically modern humans emerging from Africa.

Table 4 shows that 419Ser gene variants have spread in Eurasia compared with African population. The H1 haplotype is the most frequent among study populations. In Russians the majority haplotypes is H1 (~ 3/4). The total frequency of 419Ser haplotypes, together with H1, comprises 0.909. The European population also has high frequency 419Ser gene variants (0.933). It is likely that their ancestral population experienced a bottleneck after the Asian-European split. The lower haplotype diversity among Europeans is not only the result of purifying selection from the presumed African Asn haplotype variants, but is also due to the limited distribution of haplotypes containing the minor alleles of the rs28901637 and rs11562975 SNPs in exon 7.

The majority of Asian Asn variants contain minor alleles of the rs28901637 and rs11562975 SNPs. Specific patterns of these haplotypes are observed among Asian populations. The Asn haplotypes (H10–H17) are more frequent in Chukchi (0.501) compared with Russian (0.041) population (see Table 3). Apparently, the relatively large population differentiation, with an F_st_ value of 0.1420 ( p < 10–5) between the Russians (Europeans) and Chukchi (East Asians), emerged under the influence of the two factors: (1) the effect of purifying selection, with persistent fixation of the Ser H1 haplotype, among Russian and Kazakh ancestors in the West of Central Asia and (2) the displacement of the Asn gene variants outside of Africa by the novel Asn derivatives containing the minor rs28901637 and rs11562975 alleles of the SNPs (exon 7) among the ancestors of East Asians.

Interestingly, in all examined populations, haplotypes belonging to the H18–H22 subgroup occurred with similar frequencies (see Table 3). These gene variants include the minor alleles of three SNPs (positions 1, 4, and 5), which lead to amino acid replacements in protein. The strong LD between the minor alleles of the rs13004520 and rs17868387 SNPs (exon 7) and rs7593557 A allele (exon 11), detectable in all three populations, confirms the nonrandom character of their coevolution. Individuals carrying these haplotypes may potential produce functionally distinctive TRPM8 protein in comparison with H1 gene variant.

TRPM8 is a polymodal receptor responding to physical (cold and membrane potential) and chemical (phosphatidylinositol 4, 5-bisphosphate (PIP2), menthol and icilin) factors (Babes et al., 2011; Latorre et al., 2011). In addition, this protein functions, not only on the cytoplasmic membrane, but also on the membranes of intracellular Ca2+ stores (Bidaux et al., 2007; Mahieu et al., 2007). We performed comparative nucleotide sequence analysis of the different TRPM8 mRNA isoforms to ascertain the potential effects of their structure differences on protein function. The Figure schematically shows the structures of five TRPM8 mRNA isoforms, including the variants that encode proteins described in the literature (Tsavaler et al., 2001; Lis et al., 2005; Sabnis et al., 2008) and mRNA isoforms extracted from GenBank.

The mRNA isoform (NM_024080.1) from thermosensitive neurons of the somatosensory system encodes the full-length TRPM8 protein, which is translated from the AUG codon in exon 2 (see the Figure, a)

**Fig. 1.**
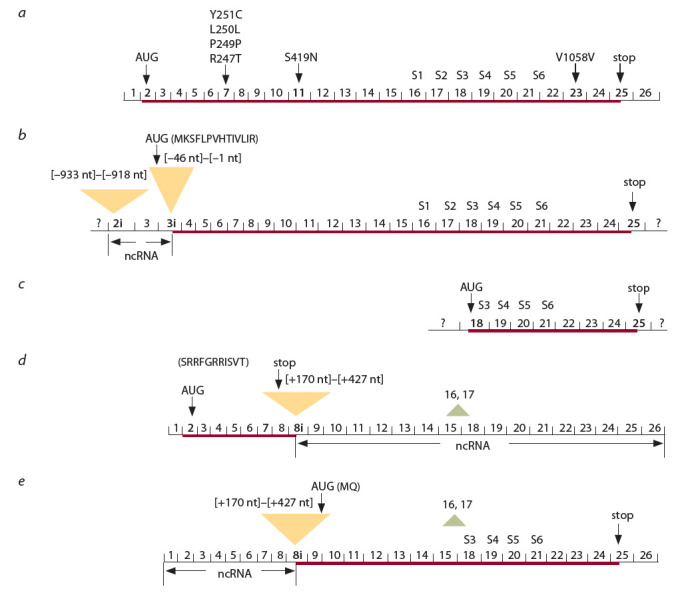
Structural features of TRPM8 mRNA isoforms. a – the reference sequence NM_024080.1 comprises 26 exons and encodes the full-length protein (1104 aa) from sensory neurons translated from the AUG codon in exon 2. The location of the transmembrane segments (S1–S6) and the studied SNPs in the mRNA are shown; b – the TRPM8-b mRNA isoform with a rearranged 5’-terminal region contain the 15 nt fragment of intron 2 (2i), exon 3, and the 46 nt sequence from intron 3 end (3i). The AUG codon is located in the 3i insert. The rearranged RNA region without AUG codon is looked as a potential 5’-UTR. The TRPM8-b mRNA region, encoded by exons 4–25, corresponds to the neuronal mRNA structure; c – the truncated ERTRPM8 mRNA isoform includes the second half of the exon 18, containing an AUG codon and the remaining part, up to exon 25, identical with neuronal mRNA isoform; d, e – the reference sequence BC143819.1 contain the 257 nt insert between positions [+171] and [+428] from the intron 8 and haven’t two exons, 16 and 17, encoding the S1 and S2 segments; d – this mRNA translates the 325 aa protein from the AUG codon in exon 2 through exons 2–8 including the 33 nts in-frame initial region of the 257 nt insert, followed by a stop codon (UGA); e – this mRNA also translates the 682 aa protein from the terminal hexanucleotide in the 257 nt insert containing a putative alternative start codon (AUG) and glutamine codon (CAG), and then through exons 9–25, without exons 16 and 17, up to UGA codon. Thus, the BC143819.1 mRNA isoform contain non-protein-coding spliced exons from the opposite parts of the translated transcript and have different AUG start codons: one in exon 2 and the other in the 257 nt insert. NcRNA – non-coding mRNA; nt – nucleotide.

(Tsavaler et al., 2001). This isoform does not contain any of the SNP minor alleles in exon 7, and has G allele rs7593557 in exon 11, consequently, the processed transcript produces the 419Ser TRPM8 protein. Hence, the mRNA (NM_024080.1) has the nucleotide sequence corresponding to H1 gene variant.

The TRPM8-b mRNA has the 5ʹ-terminal rearranged region by alternative splicing of TRPM8 pro-mRNA (see the Figure, b). This may be an indication of a change in TRPM8 protein function in prostate epithelial cells (Lis et al., 2005), compared with the neurons. The rearranged initial region in the TRPM8-b mRNA isoform contains sequences from three gene parts, including 15 nucleotides (nt) of intron 2, exon 3 (which hasn’t AUG codon), and 46 nts of the 3ʹ-terminal sequence of intron 3 including a translation start codon. Together, these parts form a specific non-coding RNA (ncRNA) region, comprising a potential 5ʹ-UTR, and potential coding sequence with the alternative AUG codon in a segment of intron 3. In consequence the TRPM8-b mRNA isoform may translates the polypeptide with truncated N-terminal domain without the peptide sequences encoded by exons 2 and 3.

The truncated ERTRPM8 mRNA isoform discovered in the bronchial epithelial cells (Sabnis et al., 2008) most likely encodes the minimum structural component required for a protein function (see the Figure, c). Exons 18 and 19 located at the 5ʹ-end of this mRNA, encode the S3 and S4 transmembrane segments, which are necessary to open the pore and activate the channel (Latorre et al., 2011; Кühn et al., 2013). It is clear, the potential-dependent sensory module of this protein (transmembrane segments S1–S4) is represented the only two transmembrane segments, S3 and S4, while the remaining S1 and S2, encoded by exons 16 and 17, are absent.

The absence of these exons is not unique, the another mRNA isoform (BC143819.1) with this feature has been identified (see the Figure, d, e). This mRNA after exon 8 contains the 257 nt insert from intron 8. This insert leads to the predicted production of two various proteins. The region comprising exons 2–8 is translated from the same start codon in exon 2 as neuronal mRNA, and ends the 33 nt sequence at the 5ʹ-end of the 257 nt insert (see the Figure, d ). Besides, the translation may starts from alternative AUG codon located in the terminal hexanucleotide of this insert (see the Figure, e). Unlike the truncated TRPM8 mRNA variant, this mRNA may potentially translates the protein having a greater extent the N-terminal domain with the peptide encoded by exon 11, including the rs7593557 SNP with the allele for serine codon. The absence of the minor alleles at rs28901637 and rs11562975 in exon 7 may indicate that both predicted proteins are alternative products of the H1 TRPM8 gene variant. It is possible that the truncated protein isoform (325 aa) influence on protein activity by interacting with the full-length TRPM8 protein, for example, via its MHRs (Melastatin Homology Regions) (Phelps, Gaudet, 2007; Pedretti et al., 2009). The mechanisms of post-translational regulation of some TRPM8 protein isoforms by others have been described in previous reports (Bidaux et al., 2007, 2012). Thus, the TRPM8 mRNAs produced by alternative splicing not only expand the protein diversity, but may also increase the range of post-translational regulation mechanisms.

The Figure illustrates a variety of TRPM8 mRNA isoforms expressed only from the H1 gene variant; however, TRPM8 mRNA isoform diversity may be far greater, given the functional coupling of the molecular machinery involved in transcription and alternative splicing (Montes et al., 2012; Kelemen et al., 2013). The results of this analysis indicate that mRNA isoforms generated by alternative splicing allow the potential synthesis of various proteins differing in the lengths of their N-terminal domains. Splicing may also generate alternative translation initiation zones, in addition to alternative mechanisms of post-translational regulation of TRPM8 activity.

From the data in Tables 3 and 4, it follows that 419Asn variants of the TRPM8 gene are more common in Asian populations compared with Russians. Their compositions are heterogeneous, probably, due to the recurrent emergence of the minor alleles of polymorphisms rs28901637 and rs11562975 (exon 7) in different 419Asn variants. It is possible that the minor alleles of these SNPs from exon 7 may influence on the features of alternative splicing of the 419Asn TRPM8 pre-mRNA and, as a consequence, the composition of mRNA isoforms in Asians. These results demonstrate the need for research of TRPM8 expression in individuals with different haplotype variants, to obtain direct confirmation of the forces underlying their selection.

## Conclusion

In summary, it appears that the prevalent fixation of the 419Asn TRPM8 gene variants carrying minor alleles SNPs in codons 249P and 250L (exon 7) in Asians is a Eurasian acquisition, which is presumably more characteristic for Eastern than Western Asians. The surrounding conditions probably favored this selection.

## Conflict of interest

The authors declare no conflict of interest.
